# Predictive mutation signature of immunotherapy benefits in NSCLC based on machine learning algorithms

**DOI:** 10.3389/fimmu.2022.989275

**Published:** 2022-09-27

**Authors:** Zhichao Liu, Guo Lin, Zeping Yan, Linduo Li, Xingchen Wu, Jingrong Shi, Jianxing He, Lei Zhao, Hengrui Liang, Wei Wang

**Affiliations:** ^1^ Department of Thoracic Oncology, The First Affiliated Hospital of Guangzhou Medical University, State Key Laboratory of Respiratory Disease, National Clinical Research Center for Respiratory Disease, Guangzhou Institute of Respiratory Health, Guangzhou, China; ^2^ Department of Thoracic Surgery, Shanghai Chest Hospital, Shanghai Jiao Tong University School of Medicine, Shanghai, China; ^3^ Department of Thoracic Oncology, Cancer Center and State Key Laboratory of Biotherapy, West China Hospital, Sichuan University, Chengdu, China; ^4^ College of Engineering, Northeastern University, Boston, MA, United States; ^5^ Tianpeng Technology Co. Ltd, Guangzhou, China; ^6^ Department of Physiology, School of Basic Medical Sciences, Guangzhou Medical University, Guangzhou, China

**Keywords:** non-small cell lung cancer (NSCLC), machine learning (ML), immunotherapy, gene, tumor mutational burden (TMB)

## Abstract

**Background:**

Developing prediction tools for immunotherapy approaches is a clinically important and rapidly emerging field. The routinely used prediction biomarker is inaccurate and may not adequately utilize large amounts of medical data. Machine learning is a promising way to predict the benefit of immunotherapy from individual data by individuating the most important features from genomic data and clinical characteristics.

**Methods:**

Machine learning was applied to identify a list of candidate genes that may predict immunotherapy benefits using data from the published cohort of 853 patients with NSCLC. We used XGBoost to capture nonlinear relations among many mutation genes and ICI benefits. The value of the derived machine learning-based mutation signature (ML-signature) on immunotherapy efficacy was evaluated and compared with the tumor mutational burden (TMB) and other clinical characteristics. The predictive power of ML-signature was also evaluated in independent cohorts of patients with NSCLC treated with ICI.

**Results:**

We constructed the ML-signature based on 429 (training/validation = 8/2) patients who received immunotherapy and extracted 88 eligible predictive genes. Additionally, we conducted internal and external validation with the utility of the OAK+POPLAR dataset and independent cohorts, respectively. This ML-signature showed the enrichment in immune-related signaling pathways and compared to TMB, ML-signature was equipped with favorable predictive value and stratification.

**Conclusion:**

Previous studies proposed no predictive difference between original TMB and modified TMB, and original TMB contains some genes with no predictive value. To demonstrate that fewer genetic tests are sufficient to predict immunotherapy efficacy, we used machine learning to screen out gene panels, which are used to calculate TMB. Therefore, we obtained the 88-gene panel, which showed the favorable prediction performance and stratification effect compared to the original TMB.

## Introduction

Immune checkpoint inhibitors (ICI) are an effective treatment for patients with advanced non-small cell lung cancer (NSCLC) (1, 2). The drugs were designed to target programmed death-ligand 1 (PD-L1)/programmed death-1 (PD-1) and disrupt inhibition of the immune response, leading to T-cell activation and restoring anti-tumor immunity (3, 4). Nevertheless, only a minority of patients with advanced NSCLC derive clinical benefits from this treatment (5). Identifying biomarkers and/or prediction models can help inform which patients would be the beneficial candidate for immunotherapy. Emerging predictive biomarkers associated with enhanced response to ICI include microsatellite instability, tumor mutational burden (TMB), PD-L1 expression, and inflammatory gene expression (6, 7). Many efforts are currently being undertaken toward improving the predictive value of different gene mutations (8). However, the routinely used prediction biomarker and/or prediction model is still not accurate enough and may not adequately utilize large amounts of medical data.

With the advent of “era of precision medicine”, the analyses of large-scale molecular data are beneficial for many aspects of oncology research, including the classification of possible subtypes, stages, and treatment of cancer (9). Accurate classification of cancers can greatly help physicians to choose the optimal treatment strategies for patients. To this end, classifying cancer into different groups is regarded as one of the most important issues in cancer therapy (10). Following the explosive growth of huge amounts of biological data, the shift from traditional biostatistical methods to computer-aided means has made machine-learning methods an integral part of today’s cancer diagnostic and prognostic prediction (11). Machine learning would certainly accelerate the progress of prediction for ICI benefits as a data-driven approach by individuating the most important features from genomic data and clinical characteristics in the current practice (12).

In this study, we exploited the potential of machine learning methods to address the issue of identifying NSCLC patients with ICI benefits. With the hypothesis that mutations in certain genes may better predict NSCLC response to ICB treatment, we aimed to develop a machine learning based-mutation signature (ML-signature) to predict ICI clinical benefits effectively.

## Methods

### Data source

This retrospective study was approved by our institutional review board (IRB No.202070). The genomic alterations and clinical data for POPLAR (NCT01903993) and OAK (NCT02008227) trials were downloaded from publicly accessible data reported by Gandara et al. (13). Both POPLAR (randomized phase II trial) and OAK (randomized phase III trial) were designed to compare single-agent atezolizumab with docetaxel as second/third-line therapy for patients with advanced NSCLC, who were unselected for PD-L1 status. In this open-label, phase 2/3 randomized controlled trial, patients with NSCLC who progressed on post-chemotherapy, Eastern Cooperative Oncology Group performance status 0 or 1 were recruited. Detailed characteristics were reported in previous reports (14, 15). Relevant data of OAK and POPLAR cohorts were provided in **Tables S1** and **S2**.

Mutations were measured by FoundationOne CDx NGS assay (16), which targets 1.1 Mb of the genomic coding sequence. As per the study protocol of POPLAR and OAK, progress-free survival (PFS) was defined as the time between the date of randomization and the date of first documented disease progression, as assessed by the investigator using RECIST v1.1, or death from any cause, whichever occurs first. Overall survival (OS) was defined as the time between the date of randomization until death from any cause. Objective response was defined as complete response and partial response according to RECIST v1.1 (17).

### ML-signature development

The ICI dataset was used as a development dataset to determine the gene signature of ICI benefits and consisted of 429 patients treated with ICI from the prospective POPLAR and OAK trials. In the research, we estimated the importance of features (mutation genes) for the ICI-benefit predictive modeling problem using the XGBoost method, which is a gradient boosting decision tree.

Using XGBoost, we developed immunotherapy benefit prediction models based on mutation features (the ML-signature) (18, 19). Gradient boosting decision tree (GBDT) methods employ an ensemble of multiple decision trees to strengthen the classification power. Each decision tree is grown by selecting the most discriminative features from the large feature candidate pool. This process relieves traditional tree-based methods from the onerous feature selection process and allows the classifier to interact directly with the features. The feature selection process of the algorithm facilitates the analysis of the features (mutation genes) that most impact the classifier and thus provides us with a method of investigating the biological mechanisms hidden within the genomic data. The higher the feature importance score of XGBoost is, the more important and effective the corresponding feature (gene) is. We obtain the top-shared ranked features (genes) based on descending order of feature importance to characterize the ML-signature. Details about the XGBoost model and code are shown in **Supplemental methods** and **Supplemental code**.

### Performance evaluation of ML-signature for immunotherapy

#### Functional enrichment and pathway analysis

For functional enrichment analysis, all genes in ML-signature were mapped to terms in the Kyoto Encyclopedia of Genes and Genomes database (KEGG) and P < 0.05 as the threshold. KEGG links genomic information with biological functions based on the online platform (https://www.genome.jp/), and KEGG results was visualized by R package ggplot2.

#### Prediction benefits stratification

ML-signature was applied to evaluate risk stratification at the individual level. The cut-off points for risk stratification of ML-signature were calculated. The ICI-benefit score was calculated as the number of mutations of the ML-signature found in a patient. We further evaluated the cut-off value for ICI-benefit score to stratify patients into ICI benefit and non-benefit groups with optimal survival stratification. Using the LOWESS smoother fitting curves, we modeled the relationship between PFS-HRs and ICI-benefit score cut-off values (20).

#### Predictive performance in comparison with TMB

Survival analyses were conducted to compare the predictive performance between ML-signature and TMB. PFS and OS were regarded as endpoints.

#### External validation

To further validate the performance of ML-signature, we evaluated its predictive power on another NSCLC dataset from external cohorts, which can be downloaded from the cBioPortal database (https://www.cbioportal.org).

### Statistical analysis

All analyses were conducted with R software (version 3.5.3) and SAS (version 9.4). A Cox proportional hazards model was used to determine the HR for survival. Kaplan–Meier methodology was used to construct the survival curves and the significances of subgroups were estimated using the log-rank test. A two-sided P <0.05 was considered a statistically significant difference.

To derive the optimal cut-offs of the candidate gene inclusion for ML-signature in the prediction of ICI-benefit/non-benefit stratification, we performed Cox regression analysis to assess the effect of inclusion criteria changes for ML-signature (the number of gene mutations was analyzed as a continuous variable) on PFS in the immunotherapy treatment arm. The curves of Wald statistic of PFS HR of different ML-signature stratification, which was determined at numbers of mutations in ML-signature, were fitted using a locally estimated scatterplot smoothing (LOESS) with a span of 0.80 (21); and structural breakpoints were then determined by Chow test (22). The Spearman’s rank coefficient was used to compare ML-signature (as a continuous variable) with TMB and SLD (sum of the longest diameter of target lesions at baseline).

## Results

**Figure 1:**
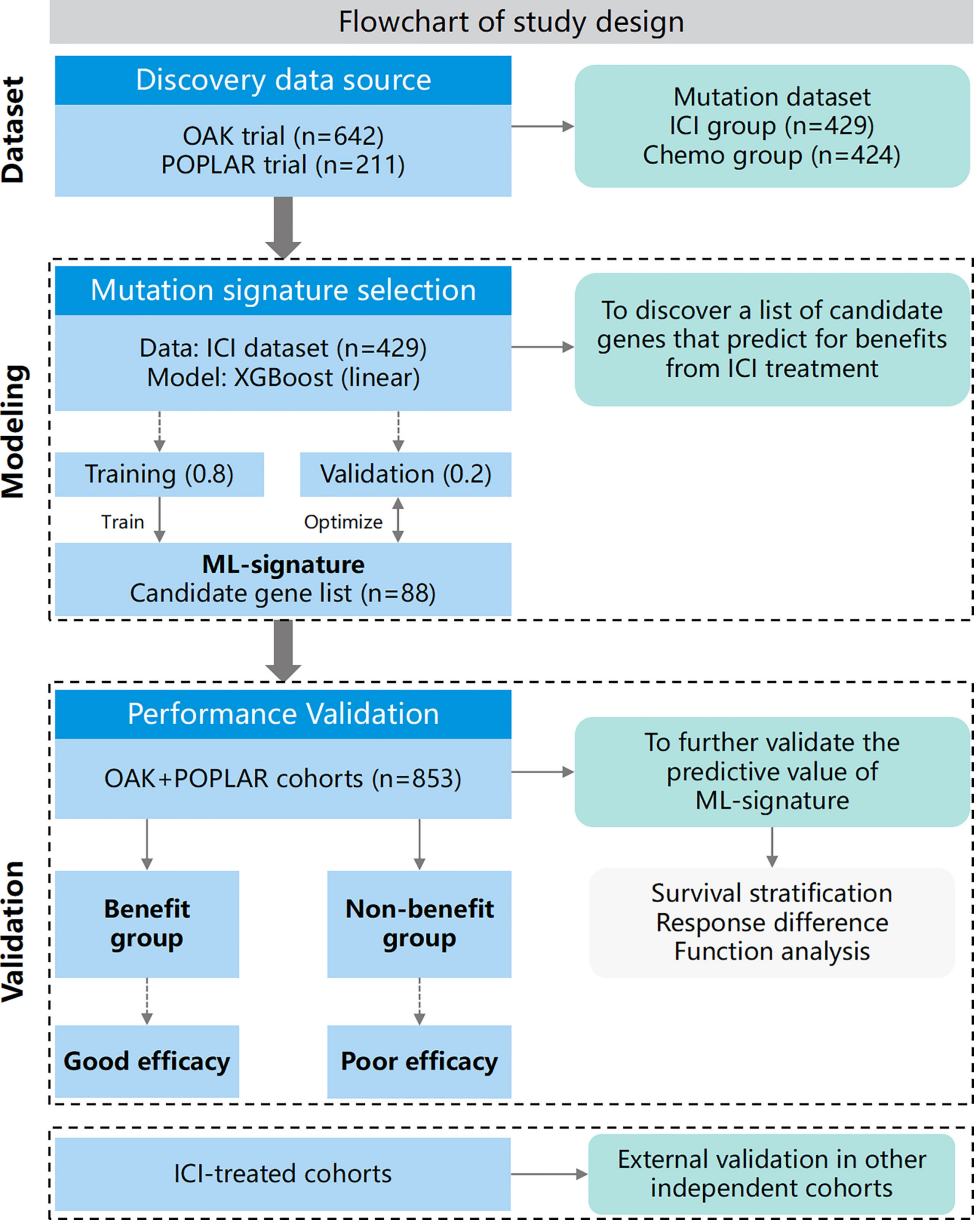
Flowchart of this study.

This study contained two sections (schematic of study design in **Figure 1**): 1.) ML-signature development, 2.) validation of the performance of ML-signature for immunotherapy efficacy.

### ML-signature construction

We hypothesized that gene mutations not only produced neoantigens but also could functionally affect the efficacy of ICI. Based on this hypothesis, we aimed to identify genes whose mutations could positively influence ICI treatment efficacy by investigating NSCLC patients from two large, published cohorts of patients treated with ICI and sequenced with FoundationOne CDx (F1CDx): 391 genes known to be involved in cancer development.

**Figure 2:**
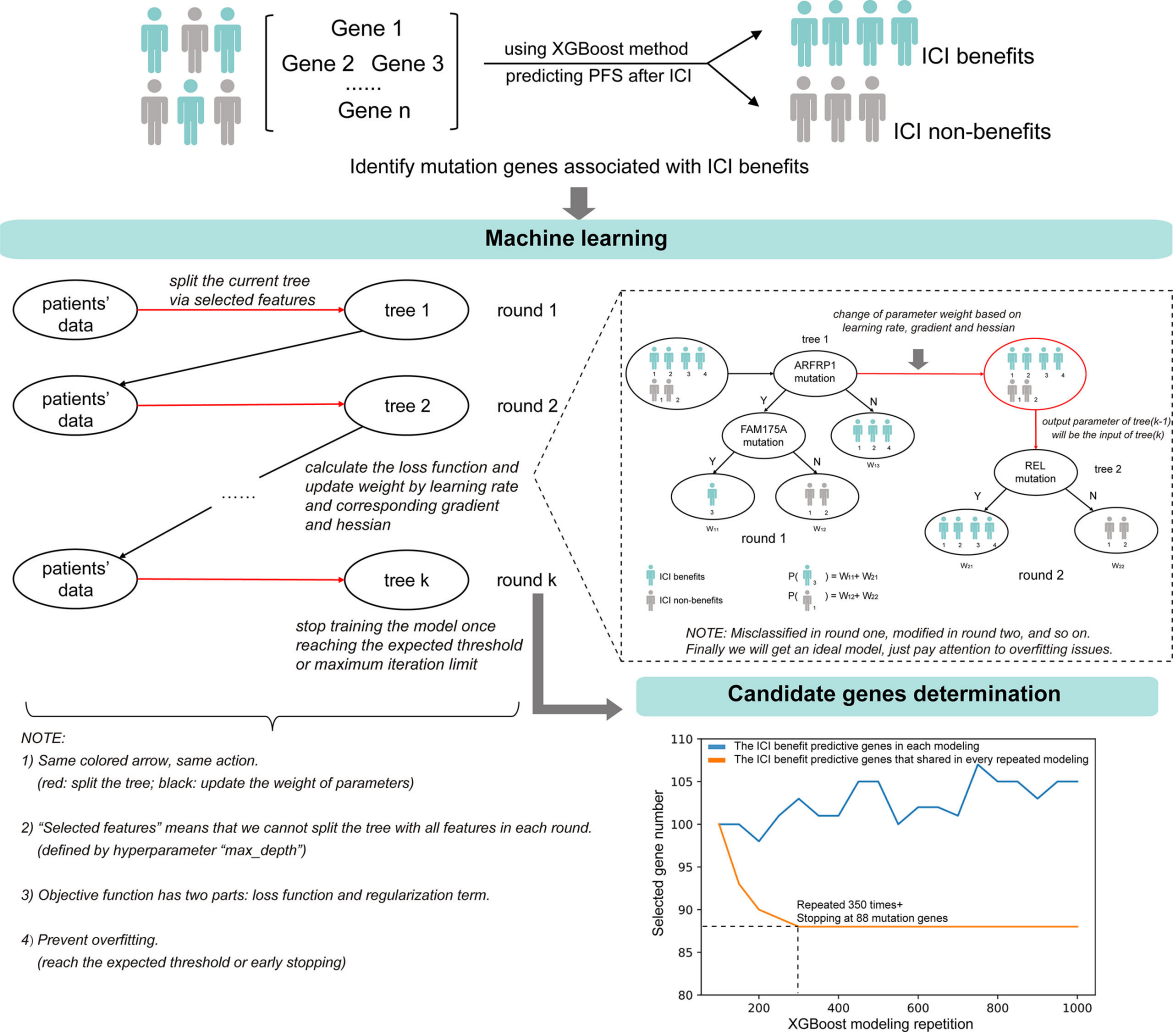
The identification of candidate mutation genes using XGBoost feature selection and the structural diagram of the machine learning model.

The ML-signature development is shown in **Figure 2**. XGBoost classification method was used to capture the importance of mutation genes for the ICI benefits. The XGBoost algorithm generates a regression model based on an ensemble of decision trees. The mutation genes are ranked based on the permutation importance method in the XGboost model. After 350 repetitions of modeling in this study, the top-ranked gene intersection between models stabilized at 88 genes. Applying these criteria to the targeted sequencing gene panel used in the study, we obtained an 88-gene panel (**Table S3**).

### ML-signature characteristics

We examined the biological functions and signal transduction pathway associated with the ICI-benefits related ML-signature. Functional enrichment (**Figures S1A, S2**) showed the immune-related pathways were significantly enriched in the ML-signature compared with those excluded genes. This result is a biological reasonableness check for the ICI-benefits gene identification using the gradient boosting decision tree analysis. Among the 853 patients with NSCLC, 467 had mutations in at least one of the 88 genes, and 243 had mutations in at least two of the 88 genes (**Figure S1B**). There was no significant difference in the distribution of mutations in 88 genes between the immunotherapy and chemotherapy groups (P>0.05).

### Prediction benefit stratification using the ML-signature

We conducted survival analyses to decide whether the ICI-benefit score could be a predictor for immunotherapy. The ICI-benefit score based on 88 genes was significant associated with favorable PFS (HR 0.755, 95%CI, 0.696-0.820, P<0.001) and OS (HR 0.849, 95%CI, 0.778-0.926, P<0.001) in patients receiving immunotherapy; while the tumor mutational burden (TMB) score was not (PFS: HR 0.991, 95%CI, 0.982-1.0, P=0.056; OS: HR 1.003, 95%CI, 0.993-1.013, P=0.572). In patients receiving chemotherapy, the ICI-benefit score was associated with poor PFS (HR, 1.061, 95%CI, 1.011-1.113, P=0.015) and OS (HR, 1.084, 95%CI, 1.030-1.140, P=0.002). These results confirmed that the ICI-benefit score based on the ML-signature was a specific predictor for immunotherapy. These data-driven results suggested an “elbow” region between≥1 and ≥6, and the cut-point analysis demonstrated that ICI-benefit score≥2 was the break point with better survival stratification in patients treated with immunotherapy (**Figure S3**). Compared to ICI-non-benefit group, ICI-benefit group showed longer PFS (HR 0.47, 95%CI, 0.38-0.57, P<0.001) and OS (HR 0.61, 95%CI, 0.47-0.78, P<0.001).

### Prediction performance of ML-signature

#### ML-signature stratified patients with favorable efficacy from immunotherapy

**Figure 3:**
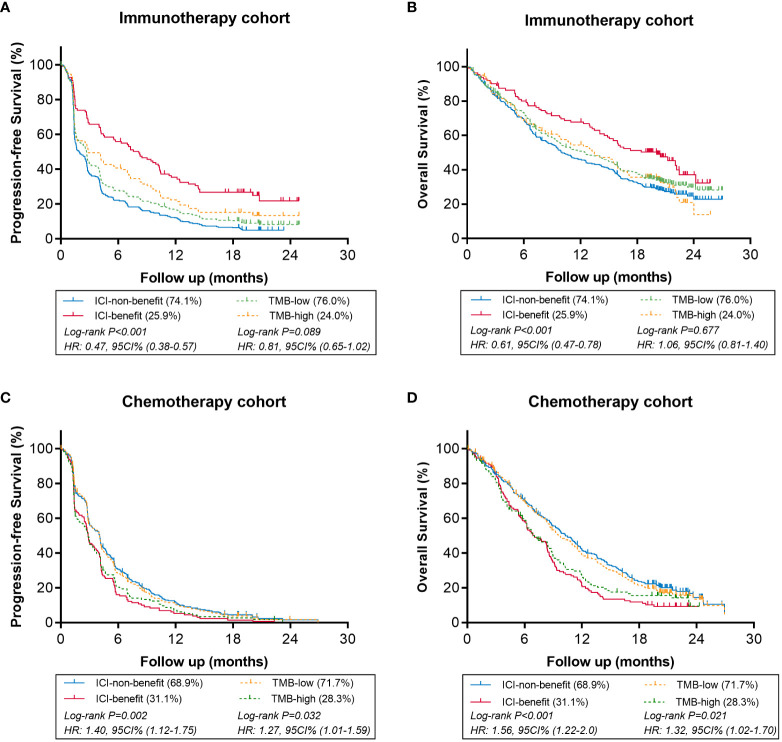
Comparison of survival in immunotherapy or chemotherapy arms using ML-signature (ICI-benefit vs. ICI-non-benefit) and TMB (high vs. low). **(A, B)** immunotherapy cohort, **(C, D)** chemotherapy cohort.

The comparison of the predictive value of modified ML-signature and TMB in immunotherapy or chemotherapy arms is presented in **Figure 3**. In patients receiving immunotherapy (**Figures 3A, B**), notably, both PFS and OS were significantly greater in the ICI-benefit group *vs*. ICI-non-group [PFS HR: 0.47 (95%CI: 0.38-0.57, P<0.001) and OS HR: 0.61 (95%CI: 0.47-0.78, P<0.001)], compared with those of TMB-high *vs*. TMB-low stratification. In patients receiving chemotherapy (**Figures 3C, D**), the ICI-benefit group was associated with poor PFS or OS [PFS HR: 1.40 (95%CI: 1.12-1.75, P=0.002); OS HR: 1.56 (95%CI: 1.22-2.0, P<0.001)], showing a greater risk stratification compared with those of TMB-high *vs*. TMB-low.

#### ML-signature associated with better predictive value for immunotherapy *vs*. chemotherapy

**Figure 4:**
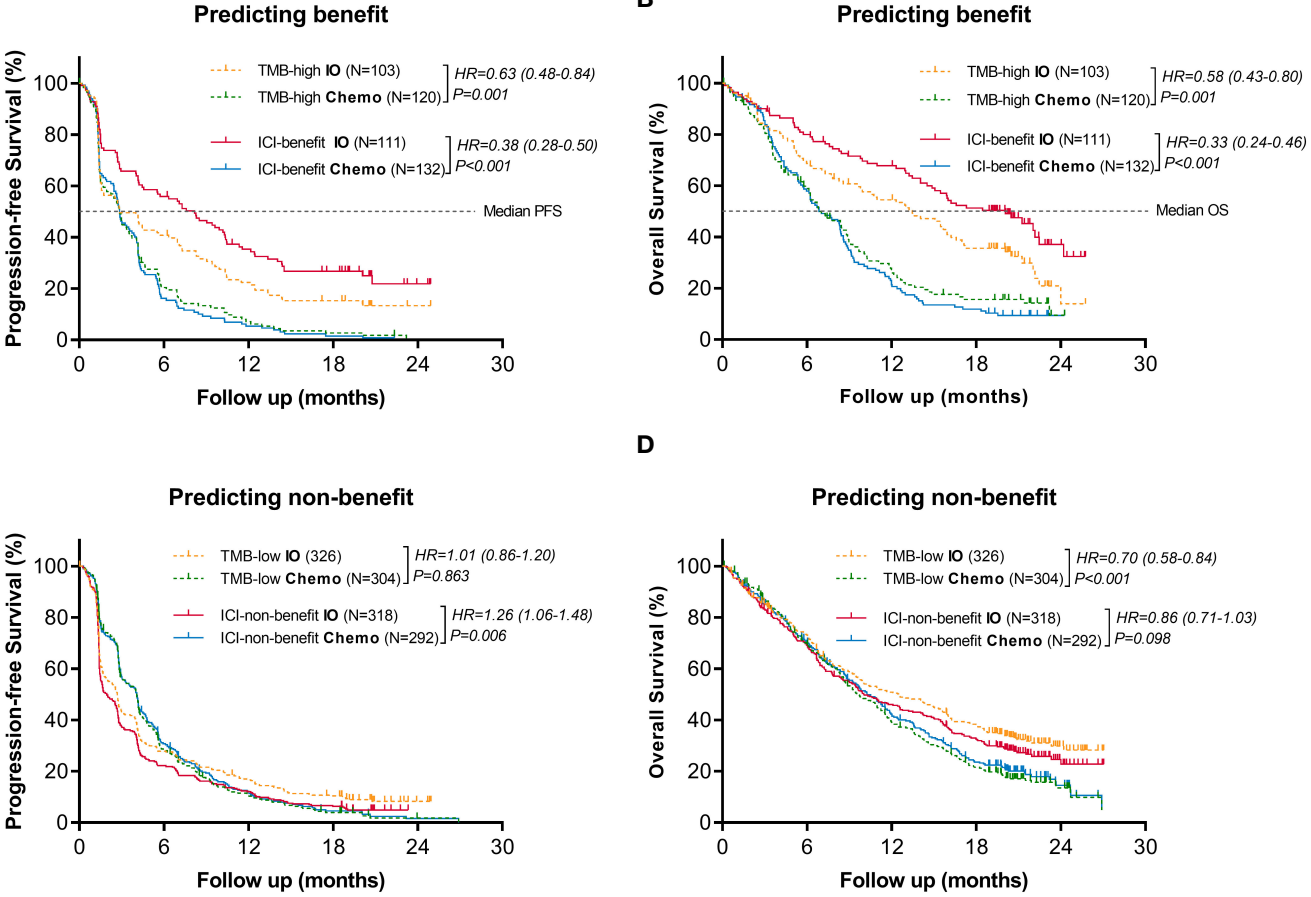
Comparison of the survival benefits from immunotherapy vs. chemotherapy between ML-signature and TMB stratification. **(A, B)** Predicting benefit cohort, **(C, D)** Predicting non-benefit cohort.

Efficacy comparison evaluating immunotherapy *vs*. chemotherapy was performed in the stratification groups based on ML-signature and TMB. When classified as benefit candidates for immunotherapy (**Figures 4A, B**), the PFS and OS benefits of immunotherapy *vs*. chemotherapy for ICI-benefit subgroup were relatively greater (HR: 0.38, 95%CI: 0.28-0.50 and HR: 0.33, 95%CI: 0.24-0.46, respectively), compared with those for TMB-high subgroup (HR: 0.63, 95%CI: 0.48-0.84; and HR: 0.58, 95%CI: 0.43-0.80, respectively). The absolute difference in median PFS and OS of immunotherapy *vs*. chemotherapy in the ICI-benefit subgroup was relatively greater (median PFS: 8.2 *vs*. 2.9 months and median OS: 20.1 *vs*. 6.9 months) compared with those of the TMB-high subgroup (median PFS: 2.9 *vs*. 2.9 months and median OS: 13.5 *vs*. 6.9 months).

Additionally, when classified as non-benefit candidates for immunotherapy (**Figures 4C, D**), results showed that the ICI-non-benefit patients benefited more from chemotherapy than immunotherapy, suggesting that stratification based on ML-signature could help to identify those who may benefit from immunotherapy. However, TMB cannot provide sufficient treatment-efficacy stratification. Overall, these results suggested immunotherapy-benefits predictive performance of the ML-signature.

#### ML-signature associated with higher objective response rate of immunotherapy

**Figure 5:**
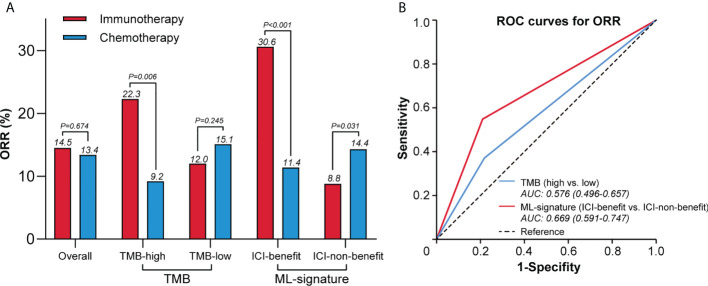
ML-signature associated with higher ORR of immunotherapy. **(A)** Difference in the ORR between ML-signature and TMB subgroups. **(B)** Receiver operating characteristic curves to predict ORR.

**Figure 5A** shows the objective response rate (ORR) in different ways of efficacy stratification. In patients classified as ICI-benefit using the ML-signature, the ORR trended towards significant benefit with immunotherapy (30.6%) *vs*. chemotherapy (11.4%). The absolute ORR benefit of immunotherapy *vs*. chemotherapy increased from 13.1% (TMB-high) to 19.2% (ICI-benefit). In the immunotherapy arm, a greater difference was observed in ORR between ICI-benefit and ICI-non-benefit subgroups (30.6% *vs*. 8.8%) compared with those of TMB-high and TMB-low subgroups (22.3% *vs*. 12.0%). Additionally, in the chemotherapy arm, the difference in ORR between ICI-benefit and ICI-non-benefit subgroups was smaller (11.4% *vs*. 14.4%) compared with the ORR between TMB-high and TMB-low subgroups (9.2% *vs*. 15.1%). In patients treated with immunotherapy, the AUC for ML-signature distinguishing between responder and non-responder patients was 0.67 (95% CI: 0.59 to 0.75), which was higher than that of TMB [0.58, 95% CI: 0.59 to 0.75)] (**Figure 5B**). Overall, these findings suggest a role for an immunotherapy efficacy-based ML-signature in promoting the identification of patients with better predictive benefits from immunotherapy.

#### Validation using external cohort

**Figure 6:**
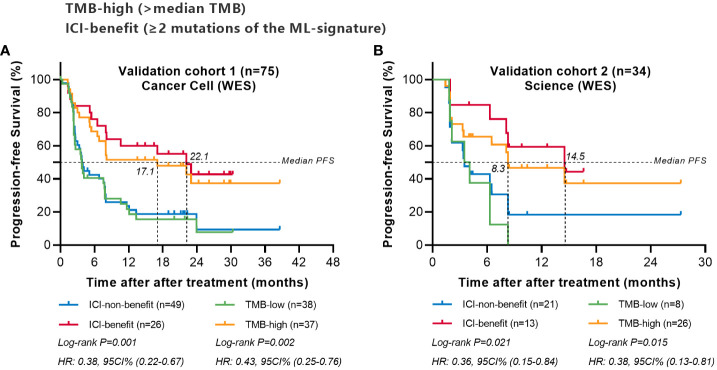
Comparing the predictive powers of ML-signature vs. TMB in patients treated with immunotherapy in external cohorts. **(A)** Cancer cell (WES) validation cohort, **(B)** Science (WES) validation cohort.

We next validate the predictive power of the ML-signature in two external cohorts of NSCLC patients treated with ICI from previously published studies (WES sequenced all patients were sequenced for gene mutations). Survival analysis of patients with different mutations in the 88-gene panel was performed. Results (**Figure 6**) revealed that patients classified as ICI-benefit candidates by our ML-signature had substantial survival advantages, which were remarkably similar to the PFS results obtained from the discovery cohort, thus validating the predictive power of the ML-signature independently.

**Figure 7:**
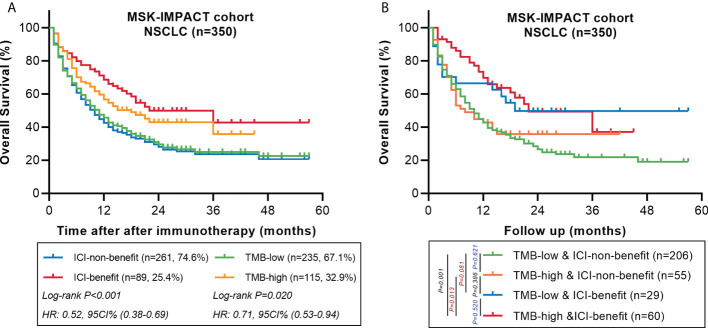
Comparing the predictive powers of ML-signature *vs*. TMB in patients treated with immunotherapy in the MSK-IMPACT cohort. **(A)** Overall survival of MSK-TMB patients with between ML-signature and TMB subgroups. **(B)** Overall survival of MSK-IMPACT patients with TMB-high and TMB-low further stratified according to groups stratified by ML-signature.

We also explored the predictive performance of the ML-signature in a cohort of 350 NSCLC patients treated with ICI from the MSK-IMPACT cohort (a large panel of targeted NGS sequenced all patients). There are 58 genes of the ML-signature (88 genes) that can be detected in the MSK-IMPACT panel (410 genes~468genes). Results also revealed that patients classified as ICI-benefit candidates by our ML-signature had better survival stratification, thus further validating the predictive power of the ML-signature (**Figure 7**).

## Discussion

The heterogeneity of cancer patients results in various therapy efficacies and deciding whether to receive ICI is momentous (23). Previous studies reported that high expression levels of PD-L1 were related to better response to ICI for NSCLC populations (24, 25). However, gene mutation also plays a crucial role in ICI for malignant cancers (26). Prediction models involving genetic mutation information are more suitable for patients with mutations. Precedented prediction models were produced based on clinical characteristics or pathological information of tumors (27), and small sample sizes restrain their generalization performance. Therefore, we constructed a mutation signature-based prediction model using machine learning to estimate the prognosis of patients treated with ICI. We demonstrated that identifying immunotherapy efficacy-based mutations could improve prediction accuracy. Our results provided a rationale for using machine learning to develop ICI-specific mutation signatures in predicting patients suitable for immunotherapy.

In this study, we identified 88 ML-signature from almost 400 genes associated with cancer progression. These ICI-benefits related ML-signature primarily enriched in immune-related signaling pathways. Immune cells and immune cytokines in the tumor microenvironment are important during cancer development, and their biological functions change dynamically with tumor progression. In addition, these ML-signature also show decreasing transduction-related signaling pathways. Signal transduction pathways involve each stage of cancer cells, from stem cells to advanced cancer cells, including proliferation, metabolism, cell cycle, DNA repair, apoptosis, differentiation, tumor extracellular matrix remodeling, angiogenesis and metastasis (28, 29). Notably, there was no significance in metabolism, DNA repair, apoptosis or other inflammatory signaling pathways. Generally, inflammation-related signaling pathways show the transduction from pro-inflammation to anti-inflammation along with tumor evolution (30). Therefore, the complex transformation of these 88 ICI-benefits related ML-signature needs further exploration. We also observed that the distributions of more than one mutation population were similar in both immunotherapy and chemotherapy groups, which indicated that patients in the chemotherapy cohort also have potential benefits from ML-signature.

Recent evidence has shown high TMB to be associated with improved clinical outcomes from ICI in multiple cancer types (31). However, TMB as a predictive biomarker for immunotherapy remains difficult to implement because tumor heterogeneity may add to the complexity of TMB analysis and lead to misestimation of the reliability of TMB prediction for immunotherapy. In this study, we hypothesized that mutations in certain genes associated with immunotherapy efficacy rather than serving as sources of roughly TMB estimation might better predict NSCLC response to ICI therapy. With the utility of ICI-benefit score developed by machine learning, we demonstrated that ML-signature provides better efficacy prediction than TMB in patients treated with immunotherapy. More mutations represent favorable survival in the immunotherapy cohort, which is consistent with the consensus that the increasing number of mutations were processed to neo-antigens and presented by major histocompatibility complex (MHC) proteins to T lymphocytes, immune-system eliminated neo-antigens (32–34). However, cancer cells impaired the activities of T lymphocytes and achieved immune escape (35). Immunotherapies blocked immune checkpoints in a targeted manner and reduced and/or re-activated T lymphocytes (36, 37).

Our ML-signature recognized the patients most likely to benefit (longer survival time and higher objective response rate) from immunotherapy. We demonstrated that immunotherapy is not suitable for all patients. Compared with immunotherapy, ICI-non-benefit patients can gain a longer survival time from chemotherapy, which can be interpreted as immune-related adverse events (irAEs). The detailed mechanisms of irAEs are still unclear. Presented mechanisms include activated T lymphocytes attacking health issues, increasing levels of autoantibodies, and inflammatory cytokines (38, 39). Although the immune checkpoint blockade is generally regarded as a tolerable treatment (40), the long-term influence of immunotherapy should be further explored.

To further validate the value of our ML-signature, we conducted survival analyses with two external NSCLC cohorts with WES data covering the overall 88 genes of ML-signature. This result validates the consistency of the internal cohort of our main result, notably the predictive effect of the ML-signature having better survival stratification than TMB, indicating that the prediction model was steady and accurate as the predictive tool for patient selection for immunotherapy. Additionally, we used the MSK-IMPACT dataset to authenticate the ML-signature, which involved more than four hundred genes and possessed the highest degree of dissemination (41). Only 58/88 genes from our ML-signature can be detected in the MSK-IMPACT dataset; similar to the above-mentioned external validation, and these 58 genes also displayed favorable stratified performance, and ML-signature can further divide into TMB high and low subgroups. To sum up, the application of these 88 genes from our ML-signature covered large-scale commercial targeted panels.

Gene mutations have also shown a crucial role in tumor heterogeneity, and the distribution of mutations represents sub-clonal status, which may compromise the efficacy of immunotherapy. The ratio of allele frequency to maximum somatic allele frequency (AF/MSAF) has also been used to represent allele frequency heterogeneity (AFH), and AFH was recognized as the negative factor of prognosis (42). To address the problems of traditional TMB in advanced patients, including insufficient tumor tissues and substandard specimens, blood-based tumor mutational burden (bTMB) emerged for clinical requirements. Dong P et al. filtered 52 candidate genes based on the Cox proportional-hazards model and demonstrated that the 52-gene panel was superior to original TMB-H (TMB ≥10) in estimating clinical benefits for ICI therapy in NSCLC patients (43). Our teams previously reported that modified bTMB had favorable performances in estimating clinical benefits from immunotherapy (44). The special characteristics of ML-signature and modified bTMB should be compared through an additional cohort. These TMB-related studies have attempted to filter genes that predict immunotherapy benefits to calculate precise TMB, and the performance of precedent models needs further improvement. Though the performance of ML-signature does not work perfectly, it showed significant progress compared to existing models (**Figure S4**) and showed sound stratification capacity. In addition, the expression level of PD-L1 is also considered to be an impartial factor in the clinical decision. We also compared ML-signature and PD-L1 level, and ML-signature showed a favorable predictive performance. We observed that only high levels of PD-L1 (TC>50% or IC>10%) had predictive value, and the ROC curves plot indicated high values for ML-signature (AUC = 0.711) but low values for PD-L1 (AUC = 0.667). Other details are shown in **Figure S5**.

There are also some limitations in our models. As a retrospective study, datasets are incomplete inevitably and information biased. Moreover, this model excluded routine clinical information, such as patients’ characteristics, pathological data, specific therapy options and surgery status. Furthermore, the partially detected genes also suggested that our model includes some genes with low predictive value and the number of eligible genes can be further refined. The ratios of eligible genes have not been tested in real-world patients. The enrichment of genes is generally shown in immune-related signaling pathways and needs further study according to their proportion in ML-signature.

## Conclusion

We provided an accurate ML-signature for NSCLC patients and demonstrated its feasibility with certain verified measurements. And our study supports the possibility and potential of using machine learning to screen for predictive molecular markers of immunotherapy efficacy or other treatments. In the ear of big data, further research can excavate novel biomarkers with the assistance of computing science and accelerate the process of translational medicine and precision medicine.

## Data availability statement

Publicly available datasets were analyzed in this study. This data can be found here: https://doi.org/10.1038/s41591-018-0134-3.

## Ethics statement

This study was reviewed and approved by the Research Ethics Committee of The First Affiliated Hospital of Guangzhou Medical University, IRB No.202070. Written informed consent was obtained from all participants for their participation in this study.

## Author contributions

ZL and GL: writing. ZY: data curation. ZL, GL, LL, XW, and JS: data analysis. ZL and GL: visualization. JH, LZ, HL, and WW: supervision. All authors contributed to the article and approved the submitted version.

## Funding

Guangzhou Medical University Discipline Construction Funds (Basic Medicine) (No. JCXKJS2022A11).

## Conflict of interest

Author JS is employed by Tianpeng Technology Co. Ltd, Guangzhou, China.

The remaining authors declare that the research was conducted in the absence of any commercial or financial relationships that could be construed as a potential conflict of interest.

## Publisher’s note

All claims expressed in this article are solely those of the authors and do not necessarily represent those of their affiliated organizations, or those of the publisher, the editors and the reviewers. Any product that may be evaluated in this article, or claim that may be made by its manufacturer, is not guaranteed or endorsed by the publisher.
