# Supplementary material for: Predictive mutation signature of immunotherapy benefits in NSCLC based on machine learning algorithms
Source: Front Immunol. 2022 Sep 27;13:989275. doi: 10.3389/fimmu.2022.989275 (PMC9552174; doi:10.3389/fimmu.2022.989275)
Supplement: Supplementary file 1 [file DataSheet_1.pdf]

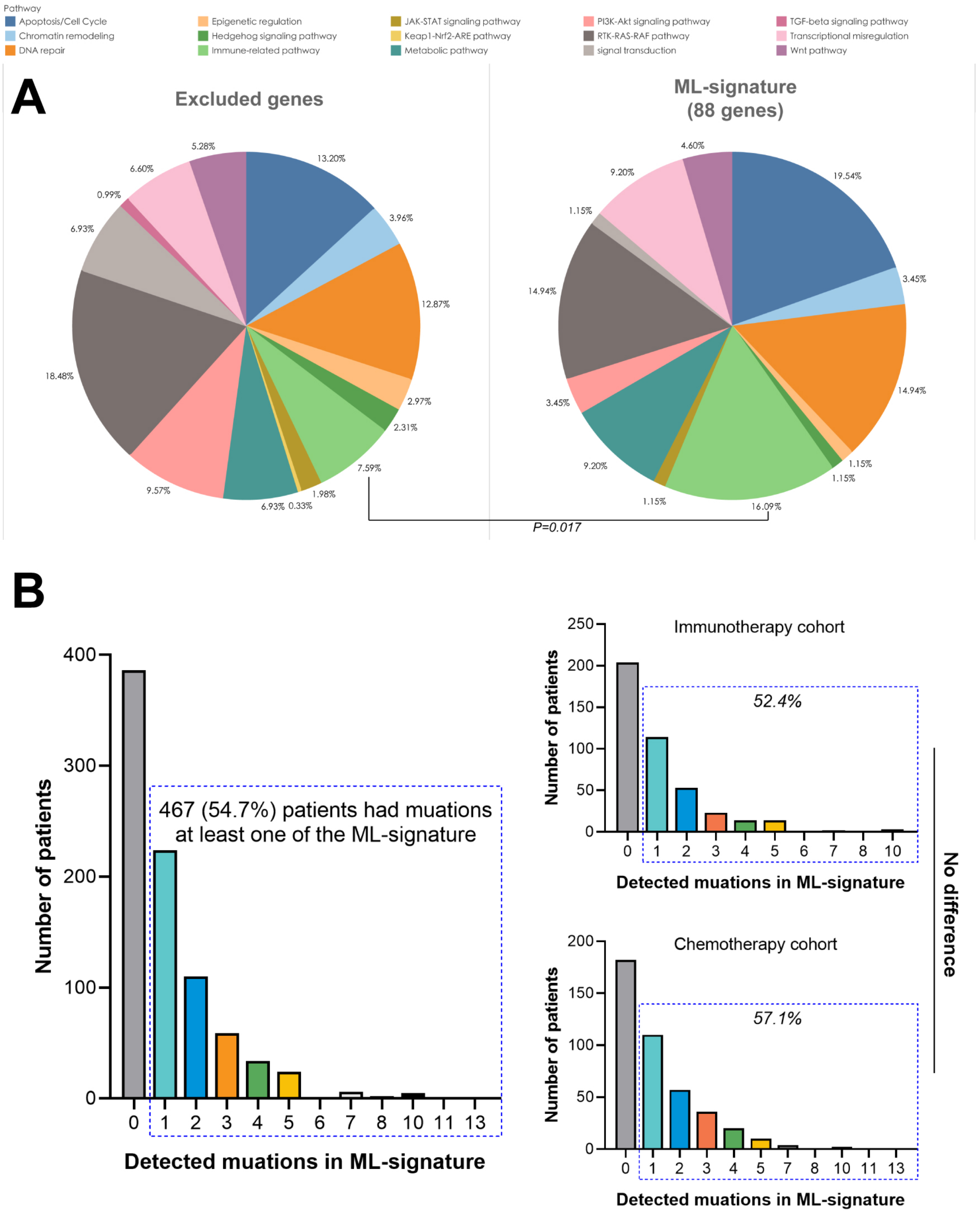

Figure S1. (A) Pathway enrichment analysis of selected 88 genes in ML-signature; (B) Distribution of mutations of the ML-signature (88 genes) among the NSCLC.
