# Supplementary figures and images for: Predictive mutation signature of immunotherapy benefits in NSCLC based on machine learning algorithms
Source: Front Immunol. 2022 Sep 27;13:989275. doi: 10.3389/fimmu.2022.989275 (PMC9552174; doi:10.3389/fimmu.2022.989275)

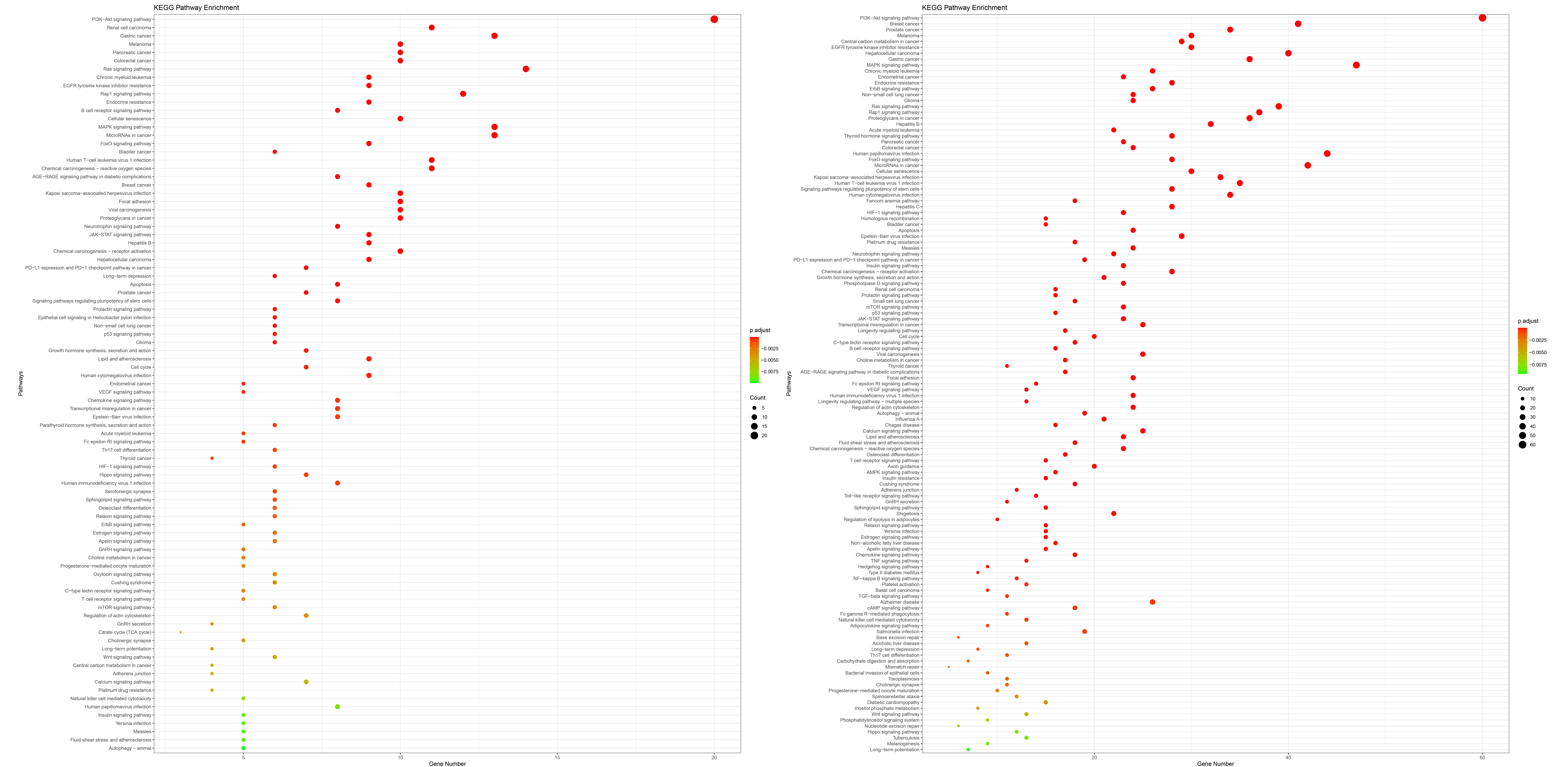

**Figure S2. The KEGG analysis of the (A) 88-gene panel and (B) excluded genes.**

Supplement: Supplementary file 2 [file DataSheet_2.pdf]
