# Supplementary material for: Predictive mutation signature of immunotherapy benefits in NSCLC based on machine learning algorithms
Source: Front Immunol. 2022 Sep 27;13:989275. doi: 10.3389/fimmu.2022.989275 (PMC9552174; doi:10.3389/fimmu.2022.989275)
Supplement: Supplementary file 3 [file DataSheet_3.pdf]

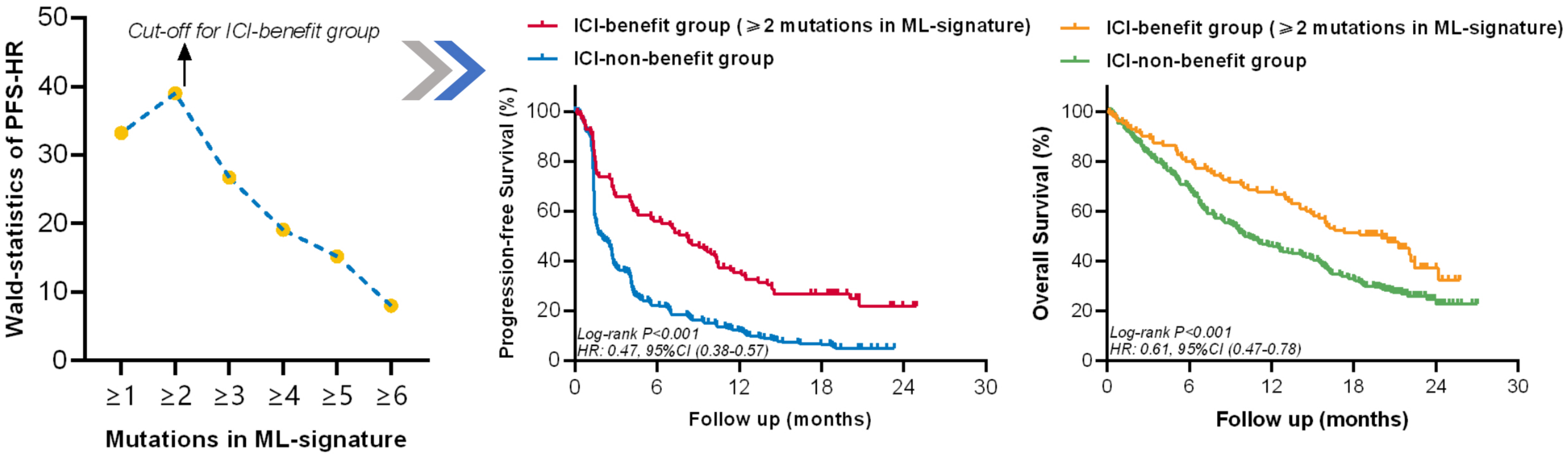

Figure S3. Determination of structural break points (cut-off) of mutations in ML-signature for the survival stratification in the ICI cohort.
