# Supplementary material for: Predictive mutation signature of immunotherapy benefits in NSCLC based on machine learning algorithms
Source: Front Immunol. 2022 Sep 27;13:989275. doi: 10.3389/fimmu.2022.989275 (PMC9552174; doi:10.3389/fimmu.2022.989275)
Supplement: Supplementary file 4 [file DataSheet_4.pdf]

A

Study	Marker	Comparison type	AUC	95% CI	Comparison type	AUC	95% CI	Reference
Gandara et al. Nat Med	bTMB	Continues variable	0.6037	0.524-0.683	high vs low	0.5765	0.496-0.657	PMID: 30082870
Wang Z, et al. JTO	LAF-bTMB	Continues variable	0.6443	0.566-0.723	high vs low	0.6212	0.540-0.702	PMID: 31843683
Liu Z, et al. JTO	Modified-bTMB	Continues variable	0.6492	0.573-0.726	high vs low	0.6175	0.535-0.700	PMID: 32340679
This study	ML-signature	Continues variable	0.7413	0.673-0.809	high vs low	0.6693	0.591-0.747	

B

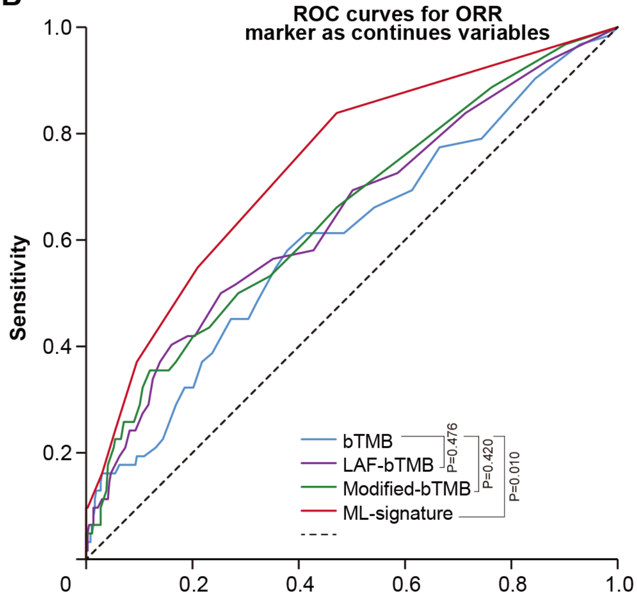

C

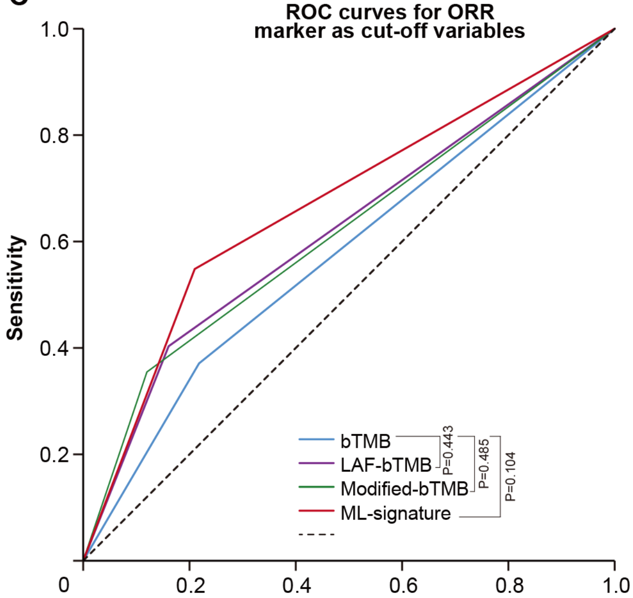

Figure S4. The predictive performance difference among various TMB and ML-signature.
