# Supplementary material for: Predictive mutation signature of immunotherapy benefits in NSCLC based on machine learning algorithms
Source: Front Immunol. 2022 Sep 27;13:989275. doi: 10.3389/fimmu.2022.989275 (PMC9552174; doi:10.3389/fimmu.2022.989275)
Supplement: Supplementary file 5 [file DataSheet_5.pdf]

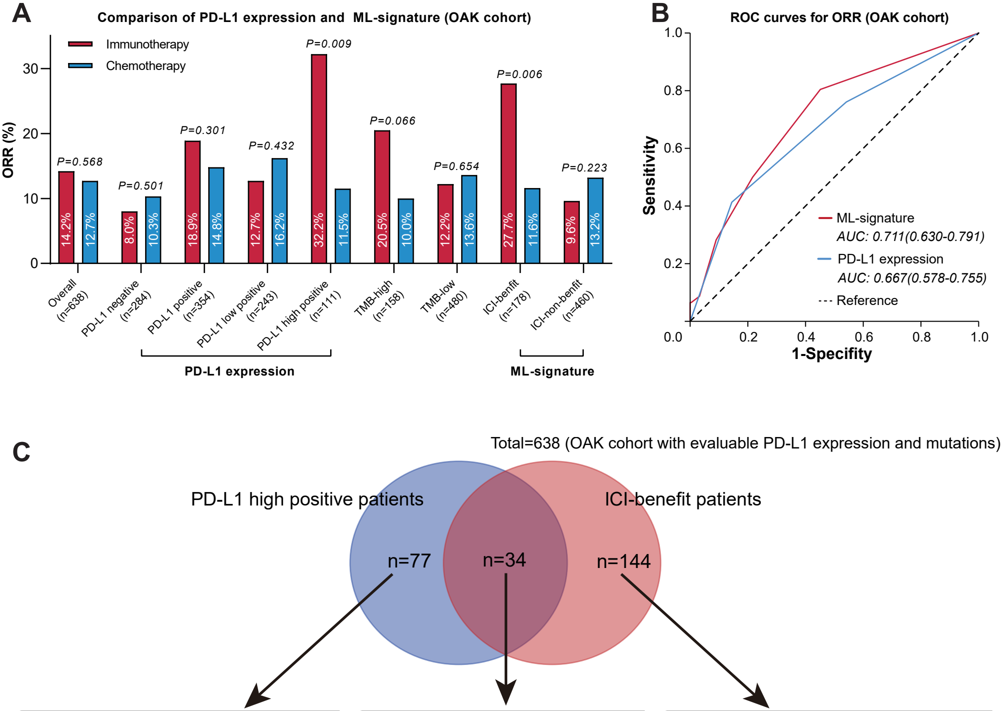

Only PD-L1 high positive n=77	
Immunotherapy (n=42)	Chemotherapy (n=35)
median PFS: 3.8m<br>median OS: 18.1m	median PFS: 3.0m<br>median OS: 9.7m
Univariate Log-rank HR<br>PFS: 0.71 (0.46-1.18), P=0.174<br>OS: 0.56 (0.31-0.99), P=0.035	
Multivariate Cox HR*<br>PFS: 0.93 (0.54-1.63) P=0.811<br>OS: 0.42 (0.21-0.84) P=0.014	
ORR	
Immunotherapy: 13/42 (31.0%)	Chemotherapy: 3/35 (8.6%)

Both PD-L1 high positive & ICI-benefit n=34	
Immunotherapy (n=17)	Chemotherapy (n=17)
median PFS: 8.5m<br>median OS: 22.2m	median PFS: 2.9m<br>median OS: 5.6m
Univariate Log-rank HR<br>PFS: 0.40 (0.18-0.86), P=0.008<br>OS: 0.23 (0.10-0.53), P<0.001	
Multivariate Cox HR*<br>PFS: 0.15 (0.04-0.53) P=0.003<br>OS: 0.04 (0.01-0.32) P=0.002	
ORR	
Atezolizumab: 6/17 (35.3%)	Docetaxel: 3/17 (17.6%)

Only ICI-benefit n=144	
Immunotherapy (n=66)	Chemotherapy (n=78)
median PFS: 7.3m<br>median OS: 16.6m	median PFS: 2.9m<br>median OS: 6.9m
Univariate Log-rank HR<br>PFS: 0.45 (0.31-0.64), P<0.001<br>OS: 0.41 (0.27-0.60), P<0.001	
Multivariate Cox HR*<br>PFS: 0.39 (0.25-0.59) P<0.001<br>OS: 0.40 (0.26-0.63) P<0.001	
ORR	
Immunotherapy: 17/66 (25.8%)	Chemotherapy: 8/78 (10.3%)

PD-L1 high positive was defined as ≥50% of tumor cell or ≥10% of tumor-infiltrating immune cell expressing PD-L1.

ICI-benefit was grouped as patients with ICI-benefit score≥2 based on the ML-signature (88 genes).

*The multivariate Cox proportional hazards model included treatment, age, sex, race, histology, ECOG status, number of prior therapy, smoking status, the longest diameter of target leisions, driver mutations and the number of metastatic sites.

Figure S5. The predictive performance difference among various PD-L1 and ML-signature.
(A) Difference in the ORR between PD-L1 expression, ML-signature and TMB subgroups.
(B) Receiver operating characteristic curves to predict ORR.
(C) Comparison of the overlap and non-overlap of PD-L1 high expression and ICI-benefit.
