# Supplementary material for: Predictive mutation signature of immunotherapy benefits in NSCLC based on machine learning algorithms
Source: Front Immunol. 2022 Sep 27;13:989275. doi: 10.3389/fimmu.2022.989275 (PMC9552174; doi:10.3389/fimmu.2022.989275)
Supplement: Supplementary file 6 [file DataSheet_6.docx]

We used the XGBoost method for immunotherapy-benefit prediction gene (feature) importance analysis.

1.1 The model was developed with all of 391 mutation genes using XGBoost method to predict the patient’s PFS (PFS = PFS time * PFS status after ICI treatment.

1.2 The analysis was performed using an 80/20 split in 429 patients’ data, in which 80% of ICI dataset was reserved for training and the remaining 20% for validation.

1.3 To avoid overfitting, we implemented the early stopping with round equal to 20 based on the cox-nloglik of the validation dataset.

1.4 We trained 100 models with different training and validation dataset, and recorded the weight of each gene in every single iteration. Then we got the respective average weight of all genes and picked those with negative weight, which means they were related to the survival of treated patients. Finally, we picked genes whose absolute value of weight was greater than the average as those with significant impact.

1.5 To avoid the bias of weight of genes from sampling, we repeated 1.4 with sampling times ranging from 100 to 1000 with interval 50. Then we observed the intersection/shared genes among different models. With the intersection/number of shared genes stabilizing, we can find the robust genes that best help to predict the patient’s PFS.
