# Supplementary material for: Predictive mutation signature of immunotherapy benefits in NSCLC based on machine learning algorithms
Source: Front Immunol. 2022 Sep 27;13:989275. doi: 10.3389/fimmu.2022.989275 (PMC9552174; doi:10.3389/fimmu.2022.989275)
Supplement: Supplementary file 7 [file DataSheet_7.docx]

import pandas as pd
import os
import numpy as np
import matplotlib as mpl
from matplotlib import pyplot as plt
from sklearn.model_selection import train_test_split
# import deepsurv
import sys
sys.path.append('/home/sjfx/huweipeng/software/DeepSurv/deepsurv')
from deepsurv.deepsurv_logger import DeepSurvLogger, TensorboardLogger
from deepsurv.deep_surv import DeepSurv
import viz
import lasagne


# event_col is the header in the df that represents the 'Event / Status' indicator
# time_col is the header in the df that represents the event time
def dataframe_to_deepsurv_ds(df, event_col='Event', time_col='Time'):
 # Extract the event and time columns as numpy arrays
 e = df[event_col].values.astype(np.int32)
 t = df[time_col].values.astype(np.float32)

 # Extract the patient's covariates as a numpy array
 x_df = df.drop([event_col, time_col], axis=1)
 x = x_df.values.astype(np.float32)

 # Return the deep surv dataframe
 return {
 'x': x,
 'e': e,
 't': t
 }


#data = pd.read_csv(sys.argv[1])
train = pd.read_csv(sys.argv[1])
#valid = pd.read_csv(sys.argv[2])
test = pd.read_csv(sys.argv[2])
model_outpath = sys.argv[3]
outfile = sys.argv[4]
#train, valid = \
# train_test_split(data, test_size=0.2, random_state=12345)

ds_train = dataframe_to_deepsurv_ds(train.drop('PtID', axis=1), event_col='PFS_status', time_col='PFS_time')
#ds_valid = dataframe_to_deepsurv_ds(valid.drop('PtID', axis=1), event_col='PFS_status', time_col='PFS_time')
ds_test = dataframe_to_deepsurv_ds(test.drop('PtID', axis=1), event_col='PFS_status', time_col='PFS_time')

hyperparams = {
 'L2_reg': 5,
# 'batch_norm': True,
 'dropout': 0.4,
 'hidden_layers_sizes': [60, 60],
 'learning_rate': 2e-04,
# 'lr_decay': 0.001,
 'momentum': 0.9,
 'n_in': ds_train['x'].shape[1],
 'standardize': False,
 'activation': 'selu'
}

# Create an instance of DeepSurv using the hyperparams defined above
model = DeepSurv(**hyperparams)

# DeepSurv can now leverage TensorBoard to monitor training and validation
# This section of code is optional. If you don't want to use the tensorboard logger
# Uncomment the below line, and comment out the other three lines:
# logger = None

experiment_name = 'predict_PFS_using_immune_gene_tmb'
logdir = '/home/sjfx/huweipeng/project/呼研院肺癌/log/tensorboard/'
logger = TensorboardLogger(experiment_name, logdir=logdir)

# Now we train the model
update_fn=lasagne.updates.adam # The type of optimizer to use. \
 # Check out http://lasagne.readthedocs.io/en/latest/modules/updates.html \
 # for other optimizers to use
n_epochs =5000

# If you have validation data, you can add it as the second parameter to the function
metrics = model.train(ds_train, ds_test, n_epochs = n_epochs, patience=5000, improvement_threshold = 0.0001, logger=logger, update_fn=update_fn)
print('Train C-Index:', metrics['c-index'][-1])
#print('valid C-Index:', model.get_concordance_index(**ds_valid))
print('test C-Index:', model.get_concordance_index(**ds_test))
train['pred_risk'] = (model.predict_risk(ds_train['x']))[:,0]
#valid['pred_risk'] = (model.predict_risk(ds_valid['x']))[:,0]
test['pred_risk'] = (model.predict_risk(ds_test['x']))[:,0]
#pred_risk = pd.concat([train,valid,test])
pred_risk = pd.concat([train,test])
pred_risk.to_csv(outfile, index=False)
viz.plot_log(metrics)
plt.savefig("/home/sjfx/huweipeng/project/呼研院肺癌/plot/learning_curve_chemo.png")
model.save_weights(model_outpath)
